# Mitigation of Insulin Resistance by Natural Products from a New Class of Molecules, Membrane-Active Immunomodulators

**DOI:** 10.3390/ph16070913

**Published:** 2023-06-21

**Authors:** Elzbieta Izbicka, Robert T. Streeper

**Affiliations:** New Frontier Labs LLC, San Antonio, TX 78240, USA

**Keywords:** insulin resistance, type 2 diabetes, natural product, membrane-active immunomodulator, diethyl azelate, drug discovery, drug development

## Abstract

Insulin resistance (IR), accompanied by an impaired cellular glucose uptake, characterizes diverse pathologies that include, but are not limited to, metabolic disease, prediabetes and type 2 diabetes. Chronic inflammation associated with deranged cellular signaling is thought to contribute to IR. The key molecular players in IR are plasma membrane proteins, including the insulin receptor and glucose transporter 4. Certain natural products, such as lipids, phenols, terpenes, antibiotics and alkaloids have beneficial effects on IR, yet their mode of action remains obscured. We hypothesized that these products belong to a novel class of bioactive molecules that we have named membrane-active immunomodulators (MAIMs). A representative MAIM, the naturally occurring medium chain fatty acid ester diethyl azelate (DEA), has been shown to increase the fluidity of cell plasma membranes with subsequent downstream effects on cellular signaling. DEA has also been shown to improve markers of IR, including blood glucose, insulin and lipid levels, in humans. The literature supports the notion that DEA and other natural MAIMs share similar mechanisms of action in improving IR. These findings shed a new light on the mechanism of IR mitigation using natural products, and may facilitate the discovery of other compounds with similar activities.

## 1. Introduction

Insulin resistance (IR) is clinically defined as the inability of a known quantity of exogenous or endogenous insulin to increase glucose uptake and its utilization in an individual as much as it does in a normal population [1]. IR is associated with conditions beyond prediabetes and type 2 diabetes (T2D), including cardiovascular and metabolic pathologies, collectively referred to as a metabolic syndrome, nonalcoholic fatty liver disease, as well as atherosclerosis, hypertension, polycystic ovarian syndrome and infectious diseases with sepsis sequelae [2,3,4,5,6,7,8]. The cause of IR is poorly understood, but the major contributors are thought to comprise systemic chronic inflammation [9] associated with abnormal lipid levels, oxidative and endoplasmic reticulum stress, insulin receptor mutations and mitochondrial dysfunctions [10].

The plasma membrane plays an essential role in cellular communication, as its structure and composition affect cellular signal transduction in health and disease [11]. We hypothesized that certain small lipophilic or amphiphilic molecules, which we have named membrane-active immunomodulators (MAIMs), affect cellular communications by modulating plasma membrane fluidity in a feedback-regulated mechanism that we refer to as adaptive membrane fluidity modulation (AMFM) [12,13].

Thanks to their membrane-fluidizing properties, MAIMs can indirectly impact the structures and functions of membrane proteins, such as receptors and ion channels. MAIMs can also directly interact with plasma membrane proteins [12,13]. In some cases, MAIMs display a nonlinear or inversed U-shaped dose response effect, which suggests their role in the maintenance of an optimal plasma membrane fluidity [13,14].

A natural product can be defined as a small molecule produced by a biological source [15]. In adherence to this definition, we have focused on single-chemical entities that were examined in pure forms or identified as active moieties in various extracts, mixtures or decoctions.

Natural products have been extensively exploited in drug development [16,17]. Some natural products comprising lipids, phenols, terpenes, antibiotics, alkaloids and gut microbiome metabolites were reported to mitigate IR, but their mode of action remains obscured. We have sought to understand how such diverse compounds can elicit comparable IR benefits. Upon the examination of the physicochemical properties of individual molecules, we realized that the mitigation of IR could be due to the “MAIM-ness” of superficially unrelated compounds.

The aim of this review is to highlight how natural products in the MAIMs family might alleviate IR through direct or indirect interactions with plasma membrane proteins. Two key players in the pathology of IR are of special interest, because their functions are affected by fluctuations in membrane fluidity [18,19,20,21]: the insulin receptor, an integral plasma membrane protein, and glucose transporter type 4 (GLUT4), which resides in insulin-responsive vesicles inside cells and shuttles between the membrane and cytoplasm [22]. Other membrane proteins, including potassium and calcium channels [23,24], ectopically expressed olfactory receptors and other G-protein-coupled receptors, implicated as players in IR [25,26,27] are also susceptible to the effects of naturally derived MAIMs.

## 2. Methods

A PUBMED search was conducted to identify peer-reviewed research articles, re-views and meta-analyses published through February 2023. The search terms included “plasma membrane” and “fluidity” or “plasticity” or “rigidity”. Secondary searches combined keywords consisting of individual chemical entities listed below and “insulin resistance” or “insulin sensitivity”, and specific pathologies, including metabolic syndrome or metabolic diseases, atherosclerosis, hypertension, polycystic ovarian syndrome, prediabetes and T2D. The collected abstracts and full papers were surveyed by the authors to confirm the relevance of the article to the topic of this review. The chemical structures were obtained from PubChem’s database (https://pubchem.ncbi.nlm.nih.gov); URL accessed on 5 June 2023. 

## 3. Results

The literature reports discussed below describe the IR-modulating effects of natural products that were selected based on their MAIM features, such as being typically lipophilic or amphiphilic small molecules, combined with reported effects of these natural products on plasma membrane fluidity, interactions with plasma membrane proteins and the modulation of biomarkers relevant for IR, as demonstrated preclinically and also preferably in the clinic. Given that MAIMs lack a single target, the structure–function relationship (SAR) used as a standard in the development of targeted drugs was not applicable here.

The representative compounds were grouped into classes of lipids, phenols, terpenes, antibiotics and alkaloids. We also added a section on gut metabolites comprising some lipids produced by gut microbiota as secondary metabolites, which, nevertheless, stood out as a separate group because of their local and systemic role within the human body.

The compounds that met the criteria of reported effects on IR mitigation and MAIM features are referenced in the text as links to the PubChem database. We presented examples of functional similarities in the regulation of IR between the lead MAIM, diethyl azelate (DEA), and other natural MAIMs.

### 3.1. Lipids

Lipids are hydrophobic or amphiphilic small molecules, including fatty acids and their derivatives, in particular esters, sterols, steroids and phospholipids.

**Figure 1:**
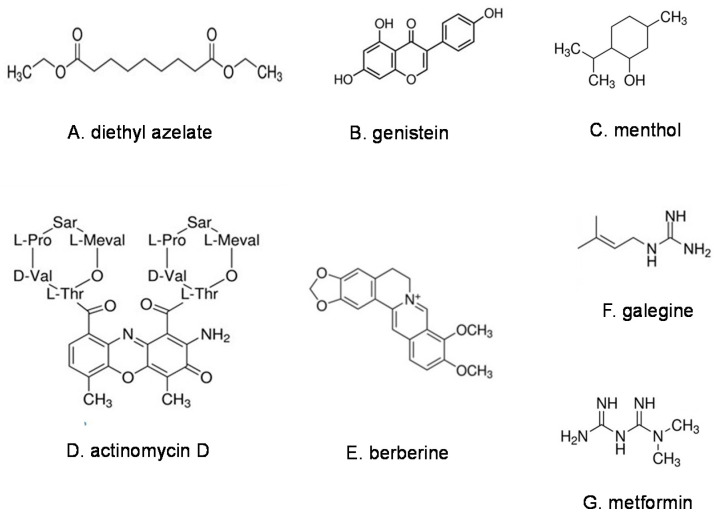
Representative natural products in the families of lipids (**A**), phenols (**B**), terpenes (**C**), antibiotics (**D**) and alkaloids (**E**–**G**).

Azelaic acid (AZA) [28] and the best characterized azelate, diethyl azelate DEA [29], (Figure 1A) are both MAIMs. Yet, as can be expected from the large differences in respective water–octanol partitioning coefficients, the respective interactions of AZA and DEA with plasma membrane are quite different [30]. DEA is a membrane fluidizer that interacts with the plasma membrane and membrane proteins, while AZA, due to its lower lipophilicity, has limited membrane interactions [12,30].

AZA and azelates occur naturally in plants, animals and humans. Archaeological records provide evidence that even ancient humans consumed AZA and its esters in the form of grains, olives, soybeans, fermented foods and alcoholic beverages [12,13,30]. Endogenous AZA produced from longer chain carboxylic acids, mainly oleic acid, is present at micromolar levels in human cerebrospinal fluid, saliva and also in breast milk [30]. The levels of AZA and azelates in the body appear to increase in response to environmental insults [31] and fasting [32].

AZA and its esters act as immunomodulators in multicellular organisms. AZA primes plant systemic immunity after infection [33]. AZA modulates the innate immune responses in human skin and induces the expression of peroxisome proliferator-activated receptor gamma (PPARγ), a key regulator of inflammation, activated by fatty acids and products of lipid peroxidation [34]. DEA and related azelates exert immunomodulatory actions in vitro and in vivo [12]. DEA is a metabolic precursor of AZA, and is rapidly converted into AZA under physiological conditions [30].

Although DEA and AZA are pharmacologically distinct entities, they share some common effects in the mitigation of IR, albeit at quite different doses. Oral AZA administered to diabetic mice at 80 mg/kg over 11–15 weeks improved glucose tolerance and decreased plasma triglycerides, glucose and cholesterol plaque formation in the arteries [35]. In a human study in overweight adult males, daily oral DEA at 1 mg/kg over 3 weeks significantly reduced fasting glucose and insulin in subjects with IR and/or hemoglobin A1c (A1c) ≥ 5.6%, and improved diagnostic lipid ratios in all cases. The impact of DEA on biomarkers of disease correlated with the degree of IR [36]. These findings are in line with the physicochemical differences between AZA and DEA. Notably, sebacic acid [37], a C10 dicarboxylic acid, purportedly has some hypoglycemic effects [38], albeit at doses nearly 100 times higher than those achieved with DEA in humans [36].

Some effects of DEA and AZA on IR may be mediated by mitochondria. Mitochondrial dysfunction has been found to be associated with obesity-induced IR and T2D [39], while the enrichment of mitochondria in skeletal muscle was reported to lower the risk of T2D [40].

AZA was reported to bind to a murine olfactory receptor Olfr544 [41]. Olfactory receptors are G-protein-coupled plasma membrane receptors that constitute over 5% of the mammalian genome. Olfr544 is widely expressed in nonolfactory murine tissues, such as the small intestine, colon, adipose tissue, liver and skeletal muscle [32]. The activation of Olfr544 with AZA stimulated mitochondrial biogenesis in mouse skeletal muscle [25,42]. In vivo treatment with AZA increased insulin sensitivity and ketone body levels [32] and upregulated genes involved in insulin signal transduction [43]. It was proposed that AZA is a fasting signaling molecule that can activate Olfr544 in various tissues [32]. In the pancreas, AZA upregulated glucagon secretion through pancreatic islets [41]. In the gut, AZA’s activation of Olfr544 increased the secretion of an insulinotropic hormone glucagon-like peptide-1 (GLP-1) [26]. Since IR and T2D are associated with the impaired postprandial secretion of GLP-1 [44,45], the endogenous activity of AZA might be beneficial in these conditions. The putative human analogue of murine Olfr544 is encoded by the OR52K1 gene, but its function, like that of many other ectopically expressed olfactory receptors, is presently unknown.

Phospholipids are the main components of cell plasma membranes, and are also canonical MAIMs. Human studies have demonstrated the association between IR and the levels of the two most abundant phospholipids, phosphatidylcholine (PC) [46] and phosphatidylethanolamine (PE) [47,48]. Interestingly, the PC/PE ratio in skeletal muscle has been found to be elevated in T2D [49]. Abnormally high or low PC/PE ratios (and even small alterations thereof) influence the mitochondrial energy metabolism and have been linked to IR and metabolic syndrome [50,51]. A U-shaped dose response of phospholipids in the control of IR was reminiscent of the dose responsiveness of some MAIMs [13].

Other lipid MAIMs, such as n-3 and n-6 polyunsaturated fatty acids (PUFAs), represented by α-linoleic acid [52] and linoleic acid [53], respectively, affect membrane fluidity upon incorporation into phospholipids [54] or as free fatty acids. The dietary n-3 PUFA counteracted IR by modulating mitochondrial bioenergetics and decreasing endoplasmic reticulum stress [55]. Higher adipose tissue levels of α-linoleic acid have been inversely associated with IR in healthy adults [56]. Rats fed a high-fat diet enriched in n-3 and n-6 PUFAs developed hyperglycemia and hyperinsulinemia, consistent with IR. The expression of insulin receptors was significantly reduced in the liver, but not in the muscle, and the n-3 PUFA diet maintained normal GLUT-4 levels in the muscle [57]. Isomers of linoleic acids activated nuclear factor kappa-B (NFκB), elevated interleukin-6 (IL-6) and induced IR in human adipocytes [58].

Cholesterol [59] is the most common steroid in human physiology and a classical lipid MAIM. Cholesterol organizes and rigidifies plasma membranes [60] where cholesterol-enriched lipid rafts regulate protein diffusion and distribution [61]. An association between IR, increased cholesterol synthesis and decreased cholesterol absorption was reported in normoglycemic men [62] and subjects with metabolic syndrome [63]. Insulin sensitivity affected the cholesterol metabolism to a greater extent than obesity [64].

Cholesterol is a precursor of primary bile salts produced by the liver. The regulation of bile acid levels is linked to the lipid and glucose metabolism. Increased plasma levels of α-hydroxylated bile acids were found to be associated with IR [65], while bile acid sequestrants reduced glucose and cholesterol levels in T2D [66,67].

As a footnote, we wanted to mention natural statins, which are not classical lipids, but inhibitors of 3-hydroxy-3-methylglutaryl coenzyme A (HMG-CoA) reductase, a key enzyme in cholesterol biosynthesis. Statins reduce systemic cholesterol with a subsequent increase in membrane fluidity. Statins are “secondary MAIMs”, which we defined as compounds that do not directly interact with the plasma membrane, but that affect the membrane composition. A natural statin, lovastatin [68], a secondary metabolite produced by fungi, was shown to modulate IR [69], presumably due to the increased signaling through the insulin receptor pathway [70].

### 3.2. Phenols

Natural phenolic compounds produced in plants are present in many foods consumed by humans [71]. Natural phenols can be viewed as MAIMs [13]. Due to their lipophilicity and usually low molecular masses, phenols can rapidly diffuse in and out of membranes and exert physiological effects promptly after absorption.

The large family of over 8000 phenolic compounds includes flavonoids and nonflavonoids. The simple nonflavonoid phenol salicylic acid [72] was recognized for the control of hyperglycemia for over a century. This activity was linked to mitochondrial uncoupling, anti-inflammatory effects mediated via NF-κB signaling and the regulation of AMP-activated protein kinase (AMPK) [73], which was reported to enhance insulin effects on GLUT4 by lowering cholesterol in plasma membranes [20]. Since salicylate is soluble in the plasma membrane [74], its aspirin-like effect [13] may explain the activity of salicylate in the control of IR. Another simple nonflavonoid, caffeic acid [75], also appears to have antihyperglycemic, hypolipidemic and hypotensive activity in vitro and in vivo [76].

Flavonoids are the most abundant polyphenols. Some flavonoids were reported to mitigate IR by affecting glucose transport and blood levels, improving insulin signaling and the function of pancreatic β-cells [77,78].

A simple flavonoid trans-chalcone [79] was shown to reduce body weight, blood glucose and insulin in healthy rats, suggesting its insulin-sensitizing activity in vivo, but the effects under IR conditions are still unknown [78].

Quercetin [80], the most abundant flavonoid in the human diet, is especially high in red onions, apples, grapes, tomatoes and dark berries. Quercetin has been shown to stimulate AMPK, increase GLUT4 translocation and protein content in skeletal muscle [81] and decrease the stiffness of plasma membranes in vitro [82].

Epigallocatechin gallate (EGCG) [83] is the most abundant catechin in green tea. EGCG decreases IR as it activates the PI3K/Akt pathway, increases GLUT4 translocation through the enhanced phosphorylation of AMPK [84] and decreases oxidative stress in vivo [85]. EGCG has showed a U-shaped dose dependence in human hepatocytes; sub-micromolar levels decreased glucose production and 5 μM of EGCG significantly increased glucose uptake, while 10 μM EGCG was toxic [86].

Luteolin [87] was reported to intercalate and disrupt bacterial cytosolic membranes [88] and significantly increase the expression of adiponectin, leptin and PPARγ in murine adipocytes in vitro [89]. Luteolin reduced obesity-associated IR in mice by activating AMPKα1 signaling in adipose tissue macrophages [90], and was suggested to modulate IR in humans [91].

Resveratrol [92] apparently targets the entire cell plasma membrane in vitro [93]. In animal and human studies, resveratrol improved markers of IR by normalizing blood glucose, reducing insulin secretion and improving insulin sensitivity. Resveratrol was also effective in managing IR-related dyslipidemia and obesity [94].

Curcumin [95] was shown to fluidize plasma membranes and decrease membrane rigidity, but also stiffened model membranes with a high cholesterol content [96,97]. Curcumin modulated the function and expression of unrelated membrane proteins in vitro [98]. In a randomized double-blind, placebo-controlled trial, daily doses of 1500 mg curcumin significantly reduced fasting blood glucose and body weight in patients with T2D [99].

Myricetin [100] present in fruits and vegetables increased membrane fluidity and reduced mitochondria dysfunction by raising mitochondrial membrane potential in vitro [101]. Myricetin also improved insulin sensitivity by restoring insulin receptors, GLUT4 expression and translocation in the muscle of rats fed a high-fructose diet [102].

Isoflavonoid fisetin [103] activated AMPK in vitro [104] and mitigated IR in vivo by blocking the inflammatory response to a high-fat diet [105].

Genistein [106] (Figure 1B), the isoflavone present at high levels in soybeans, has been recognized for its estrogenic activity. Genistein potentiated hepatic insulin signaling in rodents [107] and showed opposite effects in normal and IR mice, decreasing insulin sensitivity in the former but improving the insulin sensitivity and elevated GLUT4 translocation in the group of IR mice [108].

Human studies have yielded somewhat conflicting data on genistein. A meta-analysis of 10 randomized clinical trials involving 794 non-Asian perimenopausal or postmenopausal women showed that daily supplementation with 40 mg to 120 mg of soy isoflavones was ineffective, but comparable doses of genistein alone lowered fasting blood glucose by increasing glucose uptake in skeletal muscle [109]. Another meta-analysis of 24 clinical trials conducted in 1518 men and women who were overweight, obese or diagnosed with T2D found no significant effect of soy intake on fasting glucose and insulin levels [110].

Kaempferol [111] increased lipid organization in model membranes and facilitated the penetration of water molecules, an effect inhibited by cholesterol [112]. In vivo, kaempferol improved blood lipids and insulin levels in a dose-dependent manner, inhibited the phosphorylation of insulin receptor substrate-1 (IRS-1) and reduced the nucleic and cytosolic levels of NF-κB, tumor necrosis factor-α (TNF-α) and IL-6 levels in diabetic rats [113].

Overall, preclinical and clinical data suggest that dietary flavonoids may be beneficial for the management of IR, as they induce glucose uptake in skeletal muscle. They were also shown to decrease the production of hepatic glucose through the activation of the PI3K/Akt pathway, leading to GLUT4 translocation, the suppression of gluconeogenesis and the stimulation of glycogen production [78]. These examples underscore the MAIM effects of flavonoids.

### 3.3. Terpenes

Terpenes are a large class of over 30,000 natural compounds containing variable numbers of the isoprene unit (C5H8)_n_. Terpenes are mostly produced by plants, especially conifers and citrus trees. A distilled pine resin product, commonly used as a solvent under the name of terpentine/turpentine oil, containing a mixture of terpenes, has been recognized for its medicinal benefits for over two millennia [114].

The monoterpenes 1,8-cineole, also known as eucalyptol [115] and L-menthol [116] (Figure 1C) reportedly increased the fluidity of the outermost skin layer [117,118], acted as structural spacers in model membranes and promoted reorganization in cell membranes [119]. The chronic administration of menthol (50 and 100 mg/kg/day for 12 weeks) to mice on a high-fat diet prevented weight gain, IR, the hypertrophy of adipose tissue and the deposition of triacylglycerol in the liver. The metabolic effects of menthol in the liver and adipose tissue mirrored those of glucagon [120].

Terpenes, including limonene [121], a-terpineol [122] and 1,8-cineole, were shown to rapidly incorporate into the plasma membrane of a protozoan parasite *Leishmania amazonensis* [123]. The antidiabetic potential of multiple monoterpenes was linked to an increase in insulin sensitivity and the lowering of blood glucose and insulin levels [124].

Some triterpenes, including ursolic acid [125] and pachymic acid [126] exhibited antidiabetic and antihyperglycemic activities in preclinical studies [127]. Pachymic acid inducted the gene expression and translocation of GLUT4, the stimulation of glucose uptake and the accumulation of triglycerides in vitro [128].

A triterpene glycoside saponin [129] increased GLUT4 translocation, insulin secretion and glucose uptake in murine and human cell lines [130]. In vivo, various triterpenes increased insulin biosynthesis, secretion and signaling, decreased the levels of total cholesterol and triglycerides and lowered the body weight [127]. Terpenes from a marine algae upregulated the PPARγ gene and protein expression in the livers of diabetic rats with improved insulin sensitivity and carbohydrate metabolism [131].

D-limonene reportedly decreased blood glucose levels [132] and displayed antiatherogenic and hypolipidemic activities in rats [133]. A sesquiterpene α-cedrene [134] from cedarwood oil significantly increased the translocation of GLUT4 and increased glucose uptake in human hepatocytes in vitro, and improved glucose intolerance induced by a high-fat diet in vivo through the activation of the murine olfactory receptor Olfr16 [135]. A similar effect of α-cedrene in mice with IR was ascribed to the interaction of α-cedrene with the murine olfactory receptor 23 (Mor23) [136]. These reports suggest the possible involvement of odorant receptors in the control of IR.

Tetraterpene carotenoids exemplified by α-and β-carotene [137] are ubiquitous pigments present in photosynthetic bacteria, fungi, plants and animals. Carotenoids could modify membrane fluidity and membrane order in bacterial cells, in which carotenoid biosynthesis is integral to adaptation to cold [138]. Dietary carotenoids apparently reduce the risk of several chronic diseases, including IR, but do not prevent cardiovascular disease or cancer [139]. However, obesity and IR were shown to be inversely associated with the levels of carotenoids in serum and adipose tissue in adults, suggesting possible health benefits of dietary carotenoids [140].

### 3.4. Antibiotics

Antibiotics are broadly defined as compounds that can treat and prevent bacterial infections [141]. The classification of two major groups of antibiotics, polyketides and nonribosomal peptides, was based on their mechanism of action related to bacterial functions or growth processes.

Some broad-spectrum polyketide antibiotics, such as streptomycin [142], ampicillin [143] and vancomycin [144] were shown to worsen IR and alter gut microbiota, increasing susceptibility to metabolic syndrome and T2D [145,146]. Likewise, a monotherapy with rapamycin (also known as sirolimus) [147] exacerbated hyperglycemia, hypertriglyceridemia and the degranulation of pancreatic islets in a mouse model of T2D. Surprisingly, rapamycin combined with metformin (further discussed in Section 3.5) reversed the negative effects of rapamycin on systemic insulin sensitivity [148].

Polyketide tetracyclines from *Streptomyces* bacteria [149] can cross the plasma membrane, and their activity has been shown to be related to the transmembrane flux in human cells [150]. Tetracycline doxycycline [151] showed an unusual dose response activity in mice on a high-fat diet. Doxycycline increased their body weight at 200 μg/mL, decreased the fasting blood glucose at 20 μg/mL and increased the neogenesis of pancreatic β-cells at 2 μg/mL [152]. In a clinical case study, doxycycline induced hypoglycemia with implications for the management of IR [153].

A nonribosomal peptide actinomycin D [154] has been recognized for its antibacterial and anticancer activities ascribed to its drug–DNA interactions [155], but its interactions with cell membranes and the consequences thereof are less known. The uptake of ^3^H-actinomycin D by human cell lines in vitro was shown to vary with cell types, reflecting permeability differences in cell membranes [156]. In fungal cells, antibiotics damage the plasma membrane and induce membrane folding, cell swelling and the leakage of cellular contents [157]. In a rat feeding study, actinomycin D given before refeeding blocked the return of normal glucose-stimulated insulin secretion, despite an adequate food intake [158].

More importantly, actinomycin D (Figure 1D) has been shown to stimulate GLUT4 translocation and glucose uptake in murine adipocytes without the involvement of conventional signaling proteins and with no effect on GLUT4 endocytosis, suggesting that this antibiotic may increase the exocytosis of insulin-responsive vesicles in a process linked to the biosynthesis of RNA and protein [22].

### 3.5. Alkaloids

Alkaloids, broadly defined as natural products that contain at least one nitrogen atom, are produced by bacteria, fungi, plants and animals. The benefits of some alkaloids in the control of IR and diabetes have been attributed to the modulation of insulin signaling pathways, an improvement in the function of pancreatic β-cells and a reduction in oxidative stress and inflammation. Although most data came from preclinical studies, alkaloids are being pursued in the clinic as potential new drugs [159,160].

Emetine [161], an alkaloid present in the ipecac root, is commonly used as an expectorant, emetic and an antiparasitic treatment. Emetine inhibits ribosomal and mitochondrial protein synthesis and interferes with viral entry through host membranes [162,163]. Like actinomycin D, emetine stimulates GLUT4 translocation and glucose uptake in adipocytes [22]. The alkaloid catharanthine [164] and related alkaloids from Madagascar periwinkle (*Catharanthus roseus*) are used not only for cancer but also T2D treatments because of their antihyperlipidemic and antidiabetic effects in vivo [160].

Berberine [165] (Figure 1E), a quinoline alkaloid present in the root and bark of the berberis plant *Berberis vulgaris*, is one of the most promising alkaloids for the control of IR [166]. Berberine was shown to increase plasma membrane fluidity in rabbit erythrocytes in a dose-dependent manner. The effects of berberine on membrane proteins may explain its cardiovascular benefits [167,168].

Preclinical and clinical reports suggest the favorable activity of berberine in the treatment of T2D, obesity, hyperlipidemia, nonalcoholic fatty liver disease and gout. Berberine was shown to inhibit lipogenesis and improve insulin secretion, IR and gut microbiota disorders [169]. An insight on berberine’s mechanism of action came from the identification of a key role of the voltage-dependent K+ channel KCNH6 in the control of insulin secretion [170]. The blockade of KCNH6 channels with berberine increased insulin secretion, but only under hyperglycemic conditions, suggesting that berberine is a glucose-dependent insulin secretagogue that does not cause hypoglycemia or affects basal insulin secretion [25].

Plant-derived biguanide alkaloids are represented by galegine [171] (Figure 1F), first isolated from French lilac (*Galega officinalis*) in the 1920s following centuries of use of lilac products in folk medicine. Galegine induced weight loss and lowered blood sugar in vivo [172], but further development was overshadowed by its synthetic derivative, metformin [173] (Figure 1G). Metformin is the first-line medication for treating T2D (especially in overweight individuals), polycystic ovary syndrome, gestational diabetes and other conditions including cancer and is on the World Health Organization’s List of Essential Medicines [174,175,176,177,178,179].

Metformin’s diverse mechanisms of action are rather puzzling [180,181]. Metformin was shown to reduce hepatic glucose production, but also to play an unspecified role in the gut, exerting different effects when administered in acute versus chronic treatments, working in an AMP-dependent or AMP-independent manner and affecting mitochondrial function [180,181,182,183].

Metformin increased the membrane fluidity of human erythrocytes taken from subjects with T1D treated with the drug. When the erythrocytes isolated from these subjects were later incubated with metformin in vitro, the metformin had no further membrane-fluidizing effect, suggesting that the optimum fluidity was already attained in the body [184]. Metformin mitigated IR in rat adipocytes in vivo by enhancing the insulin-induced translocation of glucose transporters to the plasma membrane [185]. Membrane physiology has been proposed to play an unspecified role in the effects of metformin in IR and T2D [186]. Despite structural differences, metformin and berberine share similarities in the control of metabolic syndrome and T2D that may be related to common targets in the mitochondrial respiratory chain [174].

### 3.6. Gut Microbiome Metabolites: The Second Lives of Some Natural Products

The human gut hosts the largest number and species of bacteria compared to other human organs [187]. The Human Microbiome project revealed astronomical numbers (over 10^13^) of resident gut bacteria [188], which may not be commensal, but are otherwise engaged in mutually beneficial relationships. The gut microbiome and its interactions with the human body constitute a feedback loop with an impact on obesity, metabolic diseases, IR and a multitude of other conditions [189,190].

The bacterial fermentation of dietary fiber consisting of complex polysaccharides generates short-chain fatty acids (SCFAs), including acetate [191], propionate [192] and butyrate [193], which are absorbed by the host [194]. As small amphiphilic molecules, SCFAs can interact with cell plasma membranes through simple diffusion [195,196] or through active transport [197,198] due to the proton-linked monocarboxylate transporters present in the human intestinal epithelium [199].

Gut SCFAs are more than substrates for energy production, as they promote the proliferation and differentiation of the intestinal epithelium and improve the pancreatic secretion of insulin and glucagon [200]. A prospective study of 2166 participants revealed that a higher microbiome diversity was associated with fewer cases of IR, and the subjects with T2D had a lower microbiome diversity than the healthy controls, suggesting the link between the diversity and composition of the gut microbiome and the pathogenesis of T2D [201].

Secondary bile acids, including taurocholic [202], glycocholic [203], taurochenodeoxycholic [204] and glycochenodeoxycholic [205] acids, are derived from the bacterial metabolism of primary bile acids in the colon, and are viewed as potentially detrimental products [206].

## 4. Discussion

The above examples of natural products (Table 1) support the hypothesis that certain natural MAIMs can modulate IR and related pathologies. A few lipids, phenols, terpenes, antibiotics and alkaloids whose chemical structures were shown in this paper are either already used as drugs or are candidates for further development as drugs or medicinal foods/nutraceuticals for the control of IR. However, the conventional SAR analysis would likely not be capable of identifying a common denominator for such dissimilar compounds without accounting for their MAIM properties.

Taken together, historical data on the effects of natural MAIMs from in vitro and in vivo studies, anecdotal reports on human efficacy and actual clinical findings are often provocative and introduce the possibility of developing some natural MAIMs as novel modalities for the control of IR. Nevertheless, these efforts may face challenges common in the development of natural products for therapeutic applications. For example, the levels of active principles (e.g., phenols) may vary depending on the geographical location of the plant, time of harvest, processing and even time of the day [207]. Crude extracts may contain other bioactive compounds that could affect the measured endpoints because of interactions with the intended test article. So called “impurities” may be pharmacologically active or have antagonistic effects in experiments, leading to false-positive or negative results.

Even if purified and better characterized natural MAIMs and normalized quantities were to be used in assay systems, head-to-head comparisons may be difficult because of nonlinear dose responses [208] or schedule-dependent effects [178].

Another problem arises from the assay systems used to generate the data on health benefits of natural MAIMs. For in vitro assays, primary cells are difficult to standardize, while cell lines can behave differently in various testing laboratories, especially if used past the optimum number of passages [209]. In consequence encouraging preclinical efficacy of some products could not be replicated in controlled human studies [79,210].

Limited SAR preclinical–clinical correlations can be illustrated using examples of monoterpenes and flavonoids. Antidiabetic effects of monoterpenes have been ascribed to multiple pharmacological and molecular mechanisms of action, but their SARs are largely unknown [124]. In cell-free tests of flavonoids for AMPK activation, genistein was >70% less active than fisetin [104]. However, unlike fisetin [211], genistein had shown promising activity in the clinic [109]. In general, flavonoids often demonstrate impressive preclinical activities, but their clinical efficacy is limited by poor bioavailability [212].

Last, but not least, the effects of natural MAIMs may differ in healthy subjects and those with IR. For example, berberine did not alter insulin secretion under low or normal glucose conditions, but was effective in subjects with hyperglycemia [24]. Metformin reduced the incidence of T2D with the greatest benefit in the subset of subjects with higher baseline fasting glucose or A1c [213]. DEA significantly reduced fasting glucose and insulin in subjects with IR and/or A1c ≥5.6%, suggesting the correlation in DEA efficacy with the degree of IR [36].

Many natural products are MAIMs, and most are “good MAIMs” that provide health benefits, as evidenced by human use for many centuries. Natural “good MAIMs” are consumed across the world in the form of traditional Chinese medicine, Mediterranean diet and fermented foods. In contrast, relatively fewer cases of natural “bad MAIMs” are represented by oils produced by microbes, plants, animals [214] and certain gut metabolites [206].

Most “bad MAIMs” are synthetic chemicals. Notorious halogenated compounds, such as perfluoroalkyl compounds (PFASs), can readily incorporate into membranes but cannot be rapidly eliminated by the body [12,30]. Another “bad MAIM”, the endocrine disruptor 2,3,7,8-tetrachlorodibenzo-p-dioxin (TCDD) [215], alters plasma membrane function [216] and contributes to IR [217]. The induction of azelates through TCCD in humans [31] can be viewed as a chemoprotective response of the body taking advantage of the immunomodulatory role of azelates. Bisphenols are another family of “ bad MAIMs” that are of toxicological concern [218], underscored by a link between exposure to bisphenol A [219] and the elevated risk of gestational diabetes [220].

## 5. Conclusions

Certain natural MAIMs share similar characteristics in improving IR. The membrane-fluidizing potential of a natural product may be a useful criterion to consider in the development of novel functional foods or drugs for the mitigation of IR.

## 6. Patents

Some of the data on DEA pertaining to the control of IR and T2D presented in this manuscript are covered by US and worldwide patents. Representative examples of 46 patents granted to date include:

- Robert T Streeper and Elzbieta Izbicka “Azelaic acid esters in the control of insulin resistance”. US Patent No. 10251857. Application granted 4 September 2019.
- Robert T Streeper and Elzbieta Izbicka “Azelaic acid esters in the control of insulin resistance”. US Patent No. 11026912. Application granted 6 August 2021.

## Figures and Tables

**Table 1:** Examples of natural membrane-active immunomodulators (MAIMs) implicated in the mitigation of insulin resistance (IR).

Class	Example	Effects Relevant to IR Mitigation	Comments	Reference
Lipids	azelaic acid diethyl azelate sebacic acid phosphatidylcholine phosphatidylethanolamine a-linolenic acid linoleic acid cholesterol lovastatin	regulation of genes in insulin signal transduction plasma membrane fluidizer, immunomodulator GLUT4 upregulation mitochondrial energy metabolism mitochondrial energy metabolism mitochondrial energy metabolism mitochondrial energy metabolism unknown direct association reduction in systemic cholesterol level	metabolite of diethyl azelate canonical MAIM, membrane fluidizer in vitro effect canonical MAIM canonical MAIM tissue-dependent effects promotion of IR in human adipocytes canonical MAIM, membrane rigidifier statin; secondary metabolite	[12,13,28,30,31,32] [12,13,29,30,31,32,33,34,35,36] [37,38] [47,49,50,51] [47,48,49,51] [50,51,52] [53,54,56,57,58] [59,67] [68,69,70]
Phenols	salicylic acid caffeic acid trans-chalcone quercetin epigallocatechin gallate luteolin resveratrol curcumin myricetin fisetin genistein kaempferol	mitochondrial uncoupling AMPK stimulation indirect via lowering blood glucose and insulin AMPK stimulation AMPK stimulation AMPK stimulation indirect via lowering blood glucose and insulin improvement in b-cell function insulin receptor and GLUT4 expression control AMPK stimulation elevated GLUT4 translocation regulation of insulin signaling	aspirin-like membrane effect antiobesogenic effect via gut effective in healthy rats unclear antidiabetic potential U-shaped dose effect in vitro potentially effective in humans potentially effective in humans potentially effective in humans in vivo data anti-inflammatory in vivo human data in vivo data	[13,20,72,73,74] [75,76] [78,79] [80,81,82] [83,84,85,86] [87,88,89,90,91] [92,93,94] [95,96,97,98,99] [100,101,102] [103,104,105] [106,107,108,109,110] [111,112,113]
Terpenes	1,8-cineole L-menthol D-limonene α-terpineol ursolic acid pachymic acid saponin α-cedrene β-carotene	unknown direct association glucagon-like effects in liver protection against DNA damage glycation inhibition of a-amylase GLUT4 translocation GLUT4 translocation GLUT4 translocation GLUT4 translocation regulation of lipogenesis	preclinical data preclinical data preclinical data in vitro data preclinical data preclinical data in vitro data preclinical data preclinical and human data	[115,119,123] [116,119,120] [121,132,133] [122,123] [125,127] [126,128] [129,130] [134,136] [137,138,139,140]
Antibiotics	streptomycin ampicillin vancomycin rapamycin doxycycline actinomycin D	alteration of gut microbiota alteration of gut microbiota alteration of gut microbiota increased hyperglycemia induced hypoglycemia GLUT4 translocation	worsened IR worsened IR worsened IR improved IR with metformin in vivo dose-dependent in vivo in vitro data	[142,145,146] [143,145,146] [144,145,146] [147,148] [151,152,153] [154,155,156,157,158]
Alkaloids	emetine catharanthine berberine galegine metformin	GLUT4 translocation in type 1 diabetes model suppression of hyperlipidemia glucose-dependent insulin secretagogue increased weight loss, decreased blood glucose suppressed hepatic glucose production	preclinical data in vivo data preclinical and human data less potent than metformin synthetic analog of galegine	[160,161,162,163] [160,164] [165,166,167,168,169,170] [171,172] [173,174,175,176,177,178,179,180,181,182,183,184,185,186]
Gut microbiome metabolites	acetic acid propionic acid butyric acid taurocholic acid glycocholic acid taurochenodeoxycholic acid glycochenodeoxycholic acid	better pancreatic secretion of insulin, glucagon better pancreatic secretion of insulin, glucagon better pancreatic secretion of insulin, glucagon cholesterol catabolite cholesterol catabolite cholesterol catabolite cholesterol catabolite	marker of microbiome diversity marker of microbiome diversity marker of microbiome diversity potentially detrimental potentially detrimental potentially detrimental potentially detrimental	[191,194,195,196,197,198,199] [192,194,195,196,197,198,199] [193,194,195,196,197,198,199] [202,206] [203,206] [204,206] [205,206]

## Data Availability

Data sharing not applicable.
